# Insanity and Race Decay

**Published:** 1905-12-02

**Authors:** W. Lloyd Andriezen

**Affiliations:** formerly Deputy Medical Superintendent, Darenth Asylum


### Hospital Clinics.

### INSANITY AND RACE DECAY.

By W. Lloyd Andriezen, M.D.(Lond.), formerly Deputy Medical Superintendent, Darenth Asylum.

To the thoughtful mind the problem of insanity is profoundly interesting. The whole history of organic life below man can furnish no parallel to it. It is strictly of man—human.

It is, however, no isolated phenomenon in the races of man; it does not stand alone. On either side of it appear its twin-sisters, vice and crime, a horrible triad, a Cerberus which, spectre-like, looms across our view in the pathway of human progress.

The great biological advances of the century which have borne such splendid fruit in the work of Darwin on the one hand, and of Herbert Spencer on the other—have thrown a flood of light on the most varied and different branches of evolution and biology; yet singularly enough they have but little to tell us concerning the various causes and conditions of race-decay and human degenerations, problems which at the close of the century are urgently pressing for solution. Yet these causes and conditions of race-decay cannot be neglected; they are like morbid growths—cancers, veritable neoplasms in the social organism—which striking their roots deep into the race, sap the very foundations of human society.

Let me state at the outset that I do not consider *insanity*, any more than *vice* and *crime*, as mere accidents in our civilisation; comparable to the accidents and injuries to which every being is liable. For accidents and losses or destruction of property and life, or of other products of human toil, are in their nature circumscribed and limited: they mean a fixed quantity, a deficit or loss inflicted upon our bank of material or living capital, and nothing more. But the morbid entities we are dealing with now, insanity and crime, are more than that: they have a positive pathological aspect of their own, a fiendish vitality which enables them to multiply and grow beyond healthy limits, to migrate and get disseminated throughout various strata of society till the drain on the life of the social organism is too great to bear. There are two broad factors, and two only, which concur in the production of insanity and crime, and make their development possible: 1st the hereditary vices or imperfection of the organism (intrinsic cause), and 2nd the stresses of the environment (extrinsic cause), and both these factors are increasing in our day.

I will first deal with the latter—namely, stresses of the environment, because that is more obviously one the increase of which in our day is patent to even the casual observer. I think an important practical difference should be recognised between two classes of stress, which I shall, only for purposes of convenience, call (*a*) healthy and just, and (*b*) unhealthy and unjust.

I have nothing to say against the former class of stresses; they are a necessity for the evolution of life, a necessary concomitant of the increase of our population and of the increase therewith of the competitors in the race for bread and life, and of the healthy rivalries which serve to raise the standard and quality of social life to its highest efficiency; so that under such a system, and broadly speaking, our best men secure the best places. And under the same system, and taught by the lessons of religion and charity, the weak ones which are left behind in the onward march of life are not allowed to utterly perish, but they are to a great extent housed and fed and cared for. That from such healthy stresses some few should come out disorganised and deranged is a necessity born of the very laws of life, and which, therefore, is bound to occur in every form of human society. Indeed, were all such cases blotted out from the existing generation fresh contingents would arise in the next beyond a doubt. "Progress continues," says Kidd,[1] "to be everywhere marked with the same inevitable consequence of failure and exclusion from the highest possibilities of life for a large proportion of the individuals concerned."

But there exists a group of unhealthy and unjust stresses which has for so long weighed, like the proverbial mill-stone round the neck, upon a large proportion of our fellow-men, and has unfairly handicapped them at the outset, and which they are now beginning to recognise and assert as having no longer *raison d'être*. I mean those unhealthy stresses associated with early surroundings of chronic poverty and want. Early associations of vice and crime, into which—through no fault of his own—a child may be thrown; or the inability to secure by youths and girls of the slums a living wage, the frequent sequel of which is vagrancy or prostitution.

I propose next to deal with certain other aspects of the same group of causes, which have a more intimate bearing on the genesis of insanity, vice, and crime from which no proper escape has as yet been pointed out.

There is a vast and extensive class of society but a step above the very lowest; it comprises the poorer and lower classes of workers who, having no definite labour or occupation, rely upon what chance may throw in their way to gain their daily bread. Men and women in this stratum of life have always a life of chronic need and want, with the prospects of absolute destitution staring them in the face should the stresses of life increase ever so little. Mr. Charles Booth, from abundant and careful statistics in his classical work, estimates that there were three and a quarter millions of people in England in 1900 out of a population of twenty-eight millions who could not live for a week on their own resources, including a hundred thousand homeless waifs and strays, sleeping under hedgerows, bridges, carts, etc., and with no resources at all, not to mention the vast pauper populations of our workhouses and lunatic asylums. Mixed up with these, and, indeed, so intimately that it is difficult to differentiate between the two, there exists a large criminaloid, epileptic, and intemperate element. These "dwellers on the border of the swamp" have little or no idea of hygiene either of body or of mind, and in following the promptings of their untaught and all too unrestrained animal instincts, increase and multiply freely.

It is from these vast factories of degenerate humanity that there are furnished the largest numbers of our feeble-minded, crippled, or idiotic children, our epileptics, imbeciles, and moral delinquents, our petty thieves, criminals, and prostitutes. Those of them that live to attain adult life furnish afresh the larger moiety of the idiots and imbeciles, the degenerate insane, the incapables, and the criminals which fill our pauper lunatic asylums, our workhouses, and our prisons. Here it is we find all the teeming forms of mental degeneracy and the insanities gradually evolved *de novo*—*i.e.*, purely from stress in the first instance, and becoming cumulative from hereditary transmission afterwards from the mixing of similar "strains." I would here refer to the law well known to alienists and psychiatric physicians, and from observations and studies of the ancestry, causation, and heredity of both insanity and criminality, that both these morbid conditions when left to grow unchecked and to inter-breed, tend to extinction of a race in four or five generations—the last descendants of the race being sterile idiots. But before such an ultimatum can be reached, two or three intermediate generations have to be born and bred, which exhibit all the dangerous and troublesome intermediate phases of insanity, vice, and crime, and these intermediate forms are—according to local circumstances—prone to spread and get disseminated among their relatively healthier brethren, carrying the mischief with them.

The race of men found in certain districts of Switzerland, Savoy, and the Tyrol—I mean the cretins—admirably exemplifies and furnishes a striking parallel to what is here attempted to be explained—namely, how unhealthy stresses will produce brain and mind degeneration, and consequently idiocy and insanity, *de novo*. The race of the cretins is a curious and interesting one: it is restricted (in Europe) to the localities mentioned above, and its development is not due to racial but to local (telluric) causes. The condition known as cretinism is "endemic," affecting most of the population of a cretin valley. The term is in contrast to a "sporadic" cretinism which occasionally makes its appearance in presumably healthy districts where cretinism is not known to prevail. The race of cretins, then, begins with healthy ancestors who, migrating to and settling in the Cretin-producing localities, become themselves affected with the virus—variously stated to be a miasma rising from the soil by some, and a chemical condition of the drinking water by others. The virus produces in their children and grandchildren "goitre" (a disease of the thyroid gland) and a slight amount of weak-mindedness. These goitrous weak-minded descendants are known as "demi-cretins." They intermarry, and their children grow usually into fully-developed cretins; imbeciles and idiots of stunted and deformed growth in mind and body, incapable of procreating the race. Thus in the third or fourth generation the race becomes extinct, the line ending in sterile cretinoid idiocy. Yet the race of cretins is kept up, for fresh arrivals settle in these same cretin-producing localities, furnishing fresh offspring doomed to the same destiny and history.

Similarly, a slum-environment, if continued long enough, not only will produce a weak and degenerate type of offspring, but one which ultimately ends in sterile idiocy like the cretins in Nature's experiment. Passing on to the next great factor—namely, heredity, or the transmitted vices of brain organisation which the child inherits from its parents, we may say that the insanities as a whole have a distinct heredity in about 70 per cent. of cases (*Statistics of English Authors*); French writers give a slightly higher proportion, and a distinguished alienist (Dagonet in his *Traite des Maladies Mentales*, 1894) gives as high a figure as 90 per cent. Selecting now the special forms for our inquiry—namely, the alcoholic and epileptic insanities, congenital criminality, the impulsive and moral insanities of degenerates, and the tendencies to youthful intemperance, immorality, and prostitution, we find a strongly marked heredity in not less than two-thirds (65 per cent.) of the cases we have inquired into, and with more abundant and extensive statistics and sources of information this ratio tends to increase rather than diminish. No more striking, and at the same time accurate way of presenting this phase of the question can be compared with the genealogical and sociological method applied to families which exhibit the degeneracies above mentioned, as is well known to readers of The Hospital.

The classical instance, however, in history is that of the Juke's family, recorded by Dugdale, the progeny of five sisters descended from a drunken and improvident Max Juke, in the U.S.A. in 1720-40: "The progeny of these five sisters has been traced through five generations. The number of descendants include 540 who are linear blood-relations, and 169 who became related by marriage; in all 709 persons of all ages. (The aggregate probable reached over 1,000.) This vast family . . . has been on the whole a family of criminals and prostitutes, of vagabonds and paupers. Of the 709 there were 76 criminals committing 115 offences. The average of prostitution among the marriageable women down to the sixth generation was 52.4 per cent., as compared with the normal average of 1.66 per cent. in the general female population of the district." Dugdale estimates that the total cost of maintenance in prisons and penitentiaries, outdoor relief, workhouses, etc., of the Jukes family on the State of New York amounted to over a million dollars. No more instructive lesson could be laid before the physician and the medical sociologist than the above aspects of racial degeneracy variously produced by bad heredity and a noxious environment. There are reasons to think that some of the minor psychoses are strongly heritable—especially those known as the borderland or degenerative insanities (paraphrenia). Natural selection works out its inexorable law, and in the human race the problem of the accumulation, through fostering causes, of the insane, the criminal, and the degenerate will soon have to receive the serious attention of medical, medico-legal, and medico-social authorities.

[1] Social Evolution, 1902.

